# Differences in course of illness between patients with bipolar II disorder with and without epileptiform discharges or other sharp activity on electroencephalograms: a cross-sectional study

**DOI:** 10.1186/s12888-020-02968-4

**Published:** 2020-12-07

**Authors:** O. K. Drange, S. G. Sæther, P. I. Finseth, G. Morken, A. E. Vaaler, V. Arntsen, O. Henning, O. A. Andreassen, T. Elvsåshagen, U. F. Malt, E. Bøen

**Affiliations:** 1 Department of Mental Health, Norwegian University of Science and Technology, Trondheim, Norway; 2 Division of Mental Health, St. Olavs Hospital, Trondheim University Hospital, Trondheim, Norway; 3 Department of Neurology and Clinical Neurophysiology, St. Olavs Hospital, Trondheim University Hospital, Trondheim, Norway; 4 National Center for Epilepsy, Oslo University Hospital, Oslo, Norway; 5 Institute of Clinical Medicine, University of Oslo, Oslo, Norway; 6 NORMENT, Division of Mental Health and Addiction, Oslo University Hospital, Oslo, Norway; 7 Department of Neurology, Oslo University Hospital, Oslo, Norway; 8 Department of Research and Education, Division of Clinical Neuroscience, Oslo University Hospital, Oslo, Norway; 9 Psychosomatic and CL Psychiatry, Division of Mental Health and Addiction, Oslo University Hospital, Oslo, Norway

**Keywords:** Bipolar disorder, Epileptiform discharges, Sharp activity, Electroencephalography

## Abstract

**Background:**

A diagnosis of bipolar II disorder requires that the symptoms cannot be better explained by a medical condition. Epilepsy is in some cases associated with an affective syndrome mimicking an unstable bipolar II disorder. Epileptiform discharges on electroencephalograms (EEGs) are typical, but not pathognomonic, for epilepsy. A previous study has found a high frequency of epileptiform discharges and other sharp activity among patients with bipolar disorder. The aim of the study was to identify if epileptic discharges or other sharp activity per se are associated with an altered course of illness among patients with bipolar II disorder.

**Methods:**

Eighty six patients diagnosed with bipolar II disorder at two psychiatric departments were interviewed about prior course of illness and assessed with EEGs. The patients were split into two groups based on the presence (*n* = 12) or absence (*n* = 74) of epileptiform discharges or other sharp activity. Wilcoxon rank sum test, Fisher’s exact test, and Pearson’s chi squared test were used to assess differences between the groups on six variables of course of illness.

**Results:**

Patients with epileptiform discharges or other sharp activity had a history of more hypomanic episodes per year (median (interquartile range (IQR)) 1.5 (3.2) vs. 0.61 (1.1), *p* = 0.0090) and a higher hypomania:depression ratio (median (IQR) 3.2 (16) vs. 1.0 (1.0), *p* = 0.00091) as compared to patients without. None of the patients with epileptiform discharges or other sharp activity had self-reported epileptic seizures in their history.

**Conclusions:**

Epileptiform discharges or other sharp activity on EEGs are associated with more hypomanic episodes and an increased hypomania:depression ratio. Our results warrant replication in prospective studies, but suggest that EEG findings could be of prognostic importance for patients diagnosed with bipolar II disorder in psychiatric care.

**Trial registration:**

ClinicalTrials.gov (NCT00201526).

**Supplementary Information:**

The online version contains supplementary material available at 10.1186/s12888-020-02968-4.

## Background

A diagnosis of bipolar II disorder requires a history of at least one hypomanic and one depressive episode. These episodes should not be attributable to a medical condition [1]. At least 0.5–1% [2, 3], but probably more [4], of the general population fulfill the diagnostic criteria of bipolar II disorder.

Electroencephalograms (EEGs) capture signals from synchronized electrical activity in the synapses of cortical pyramidal cells [5]. Some signals, like spikes, sharp waves, and spike-waves, are classified as epileptiform discharges [6, 7]. Detection of epileptiform discharges are supportive for the diagnosis of epilepsy [8]. Epilepsy is associated with affective syndromes which could be classified according to their relation to the ictus of seizures [9, 10]. One of these syndromes, the interictal dysphoric disorder, has a course of illness mimicking an ‘unstable bipolar II disorder’ [11].

Epileptiform discharges can occur in the absence of seizures and a diagnosis of epilepsy [8, 12]. The prevalence of epileptiform discharges in samples without epilepsy ranges from 0.5% among healthy flight personal [13] to 4% in a tertiary neurological center [14]. Further, in a study of 202 patients hospitalized due to manic episodes, 5% had epileptiform discharges and 12% had other sharp activity on EEGs [15].

A question of clinical relevance is whether epileptiform discharges or other sharp activity per se are associated with an altered course of illness among patients diagnosed with bipolar II disorder in psychiatric care. Levy et al. have previously reported bitemporal paroxysmal sharp waves in three out of five patients with rapid cycling bipolar disorder [16]. Among 20 patients with treatment resistant bipolar disorder, Cole et al. found temporal lobe abnormalities in five out of 13 patients assessed with EEGs [17]. None of these studies were conducted on samples of patients with bipolar II disorder with comparable control groups.

Here, we compare the course of illness in patients with bipolar II disorder with and without epileptiform discharges or other sharp activity. Because of the scarcity of prior information on the topic, we conducted an exploratory study investigating six variables on course of illness.

## Methods

### Study design

The Bipolar Research and Innovation Network Norway (BRAIN)-study is a multi-center cross-sectional study aiming to describe the spectrum of patients with bipolar disorder in a clinical setting (ClinicalTrials.gov NCT00201526).

### Setting

Norway has a universally available, publicly financed (the patient’s charge is restricted to ~ 260 USD per year), and catchment area based health care system. For the present study, we used data from two psychiatric departments that had access to original EEG descriptions recorded around the time of inclusion to the BRAIN-study.

The Department of Østmarka, St. Olavs Hospital, Trondheim University Hospital included participants from 2003 through 2010 from its outpatient bipolar disorder clinic and acute psychiatric ward. Participants were referred to EEGs as part of the clinical work-up before inclusion or, if not already referred and residing within reasonable travel distance, at inclusion. Recording and assessment of EEGs were conducted at the Department of Neurology and Clinical Neurophysiology, St. Olavs Hospital, Trondheim University Hospital.

The Department of Psychosomatic Medicine, Oslo University Hospital included participants from 2008 through 2010 from psychiatric outpatient clinics in the Oslo area. Participants were referred to EEGs at inclusion. Recording and assessment of EEGs were conducted at the National Centre for Epilepsy, which is a department at the hospital.

### Participants

Patients diagnosed with bipolar disorder aged ≥18 years were invited to participate in the BRAIN-study. The only exclusion criterion was inability to provide informed, written consent. We do not have data on the number of patients eligible for the BRAIN-study.

Eighty-six participants with bipolar II disorder and available original EEG descriptions recruited from the Department of Østmarka (Sample 1; *n* = 64) and the Department of Psychosomatic Medicine (Sample 2; *n* = 22) were included in the present study.

### Data sources/measurements

Diagnoses of bipolar II disorder were made by trained clinicians according to the Structured Clinical Interview for DSM IV Axis I Disorders (SCID I) [18] in Sample 1 and the Mini-International Neuropsychiatric Interview (M.I.N.I.) [19] in Sample 2. In addition, the Hypomania Checklist (HCL-32) [20] was used in Sample 2 to strengthen the assessment of hypomanic symptoms.

All participants were assessed with a Norwegian adaptation of the Network Entry Questionnaire (NEQ). The NEQ is a semi-structured interview developed by the Stanley Foundation [21] covering sociodemographic factors, family history of mental disorders, medical history, pharmacological treatment, and course of illness. The Inventory of Depressive Symptoms Clinician Rated 30 (IDS-C_30_) [22] and the Young Mania Rating Scale (YMRS) [23] were used to determine the degree of affective symptoms at inclusion.

Clinical neurophysiologists received a standard referral to EEGs in Sample 1 (i.e. were not necessarily blinded for information from the clinical assessment) and a minimal referral in Sample 2 (i.e. were blinded for information from the clinical assessment). Scalp EEGs were recorded and assessed according to clinical protocols used at the respective departments at the time of inclusion (see Supplementary notes 1a and 1b for details). The NicoletOne EEG system (Natus Medical Inc., Ca, USA) with a sampling rate of 256 Hz was used in both samples. Electrode placements were according to the 10–20 and the 10–10 system in Samples 1 and 2 respectively. Total recording times were 20 and 90 min in Samples 1 and 2 respectively. Activation procedures included hyperventilation and photo stimulation in both samples. In both samples clinical neurophysiologists visually interpreted the EEGs and wrote a descriptive note in the medical records.

### Variables

#### Sample characteristics

We used self-reported variables from the NEQ to describe sociodemographic factors, family history of mental disorders, medical history, and pharmacological treatment. Years of education was defined as the number of years at upper secondary schools, university colleges, and universities. A family history of schizophrenia, bipolar disorder, and depression was defined positive for each disorder if the diagnosis had previously been made in one or more 1st degree relative(s). A medical history of migraine, traumatic brain injury, and epileptic seizures was defined positive if the participant reported a previous diagnosis of the conditions. Pharmacological treatment referred to current treatment of an agent within each class (i.e. lithium, anticonvulsants, antipsychotics, antidepressants, or anxiolytics/hypnotics). Psychiatric comorbidity was assessed with the SCID 1 in Sample 1 and the M.I.N.I. with some modules from the M.I.N.I. plus (e.g. ADHD) in Sample 2. Current affective symptom burden was defined as total scores on the IDS-C_30_ and the YMRS.

#### Course of illness

All variables on course of illness were derived from the NEQ. Age at onset was defined by the age of first symptoms of a depressive or hypomanic episode as reported in the NEQ and verified by the SCID 1 in Sample 1 and the M.I.N.I. in Sample 2. Depressive and hypomanic episodes per year were defined as the fractions of the total number of episodes above the duration of illness. The hypomania:depression ratio was defined by the fraction of the total number of hypomanic episodes above the total number of depressive episodes. Admissions per decade was defined by admissions due to depressive episodes divided on duration of illness and multiplied by ten. Rapid cycling was defined by four or more episodes per year during lifetime.

#### Epileptiform discharges and other sharp activity

EEG descriptions in the BRAIN-study are categorized into one of the following five categories as defined by the NEQ: 1) *normal*, 2) *slow waves not further specified / unspecific pathology / mild dysrhythmia not further specified*, 3) *asymmetric theta*, 4) *delta waves*, or 5) *spike-waves*.

We retrospectively reviewed the original EEG descriptions for all participants and specified the type and location of epileptiform discharges or other sharp activity. Descriptions of spikes, sharp waves, spike-waves, or sharp-and-slow waves were classified as epileptiform discharges [6, 7]. In cases where clinical neurophysiologists used Norwegian terms which translate to “sharp potentials” and “sharp waves”, but not explicitly used the English terms spikes, sharp waves, spike-waves, or sharp-and-slow waves, we could not be certain whether the graphoelements fulfilled the established criteria of epileptiform discharges [6, 7]. Thus, we choose to classify these descriptions as other sharp activity.

### Bias

We repeated our analyses of variables representing course of illness in Samples 1 and 2 to indirectly test if our results could be explained by systematic differences between the sub-samples (e.g. in routines for clinical assessments, and EEG recordings and assessments).

The reliability of self-reported answers in the NEQ were evaluated by clinical judgement on a four point ordinal scale ranging from 1 (‘very reliable’) to 4 (‘unreliable’).

### Study size

The study size was determined by the number of patients who accepted to participate and fulfilled the inclusion criteria.

### Statistical methods

The statistical software R version 3.4.3 [24] and RStudio version 1.1.383 [25] were used for all calculations. We split the sample based on the presence or absence of epileptic discharges on EEGs. Groups were compared on six variables representing course of illness. We also conducted separate analyses in each of the two sub-samples.

Fisher’s exact test or Pearson’s chi squared test and the Wilcoxon rank sum test were used for categorical and non-normally distributed continuous variables, respectively. The significance level was set at *p* < 0.05. Due to the exploratory study design, no correction for multiple testing was applied.

## Results

Eighty-six participants with bipolar II disorder and available original EEG descriptions were included in the present study. Mean age was 37 years (SD ±12) and 56% were women. Twelve participants (14%) had epileptiform discharges or other sharp activity.

### Sample characteristics

The groups with and without epileptiform discharges or other sharp activity did not differ substantially on variables of sociodemographic factors, family history of mental disorders, medical history, psychiatric comorbidity, pharmacological treatment, or symptom burden at inclusion (Table 1, Supplementary Tables 1a and 1b).

**Table 1:** Characteristics of participants with and without epileptiform discharges or other sharp activity

	ED/SA+ (*n* = 12)	ED/SA- (*n* = 74)	p-value
Age			0.29^†^
mean ± SD	35 ± 14	38 ± 12	
median (IQR)	32 (9.3)	38 (20)	
Women, n (%)	8 (67)	40 (54)	0.62^§^
Years of education^a^			0.23^†^
mean ± SD	5.8 ± 3.1	4.7 ± 3.3	
median (IQR)	6.0 (3.0)	4.0 (4.0)	
Family history, n (%)			
Schizophrenia^b^	0 (0)	5 (7.1)	1.0^‡^
Bipolar disorder^c^	3 (25)	13 (18)	0.69^‡^
Depression^d^	5 (42)	39 (53)	0.66^§^
Medical history, n (%)			
Migraine^e^	3 (25)	11 (15)	0.41^‡^
Traumatic brain injury^e^	1 (8.3)	11 (15)	1.0^‡^
Epileptic seizures	0 (0)	3 (4.1)	1.0^‡^
Pharmacological treatm., n (%)^f^			
Lithium	0 (0)	2 (2.7)	1.0^‡^
Anticonvulsants	8 (73)	62 (84)	0.40^‡^
Antipsychotics	4 (36)	29 (39)	1.0^‡^
Antidepressants	5 (45)	34 (46)	1.0^§^
Anxiolytics/hypnotics	3 (27)	24 (32)	1.0^‡^
None	1 (9.1)	2 (2.7)	0.34^‡^
Psychiatric comorbidity, n (%)			
Alcohol harmful use or dependence	0 (0)	10 (14)	0.34^‡^
Substances harmful use or dependence	0 (0)	4 (5.4)	1.0^‡^
Agoraphobia	0 (0)	3 (4.1)	1.0^‡^
Social phobia	1 (8.3)	8 (11)	1.0^‡^
Specific phobia	1 (8.3)	0 (0)	0.14^‡^
Panic disorder	3 (25)	12 (16)	0.43^‡^
Obsessive-compulsive disorder	1 (8.3)	1 (1.4)	0.26^‡^
Attention deficit hyperactivity disorder ^g^	0 (0)	1 (5.9)	1.0^‡^
Symptom burden			
IDS-C_30_ score^d^			
mean ± SD	23 ± 13	25 ± 10	0.49^†^
median (IQR)	25 (12)	25 (17)	
YMRS score			0.77^†^
mean ± SD	1.8 (2.3)	1 (2.5)	
median (IQR)	2.3 (3.0)	0.5 (4.0)	

Abbreviations: *ED* epileptiform discharges, SA other sharp activity, IDS-C30 Inventory of Depressive Symptoms Clinician Rated, IQR interquartile range, *SD* standard deviation, *YMRS* Young Mania Rating Scale. Missing data: ^a^ 1 in ED/SA- group, ^b^ 4 in ED/SA- group, ^c^ 2 in ED/SA- group, ^d^ 1 in ED/SA- group, ^e^ 1 in ED/SA- group, ^f^ 1 in ED/SA+ group, ^g^ 7 in ED/SA+ and 57 in ED/SA- group (SCID 1 used in Sample 1 does not assess ADHD). † Wilcoxon rank sum test, ‡ Fisher’s exact test, § Pearson’s chi squared test

None of the participants with epileptiform discharges or other sharp activity had a history of epileptic seizures as compared to three in the control group. The majority of participants in both groups used anticonvulsants (8/11 with epileptic discharges or sharp activity (missing data for one participant) vs. 62/74 without epileptic discharges or sharp activity, *p* = 0.40).

### Course of illness

**Fig. 1:**
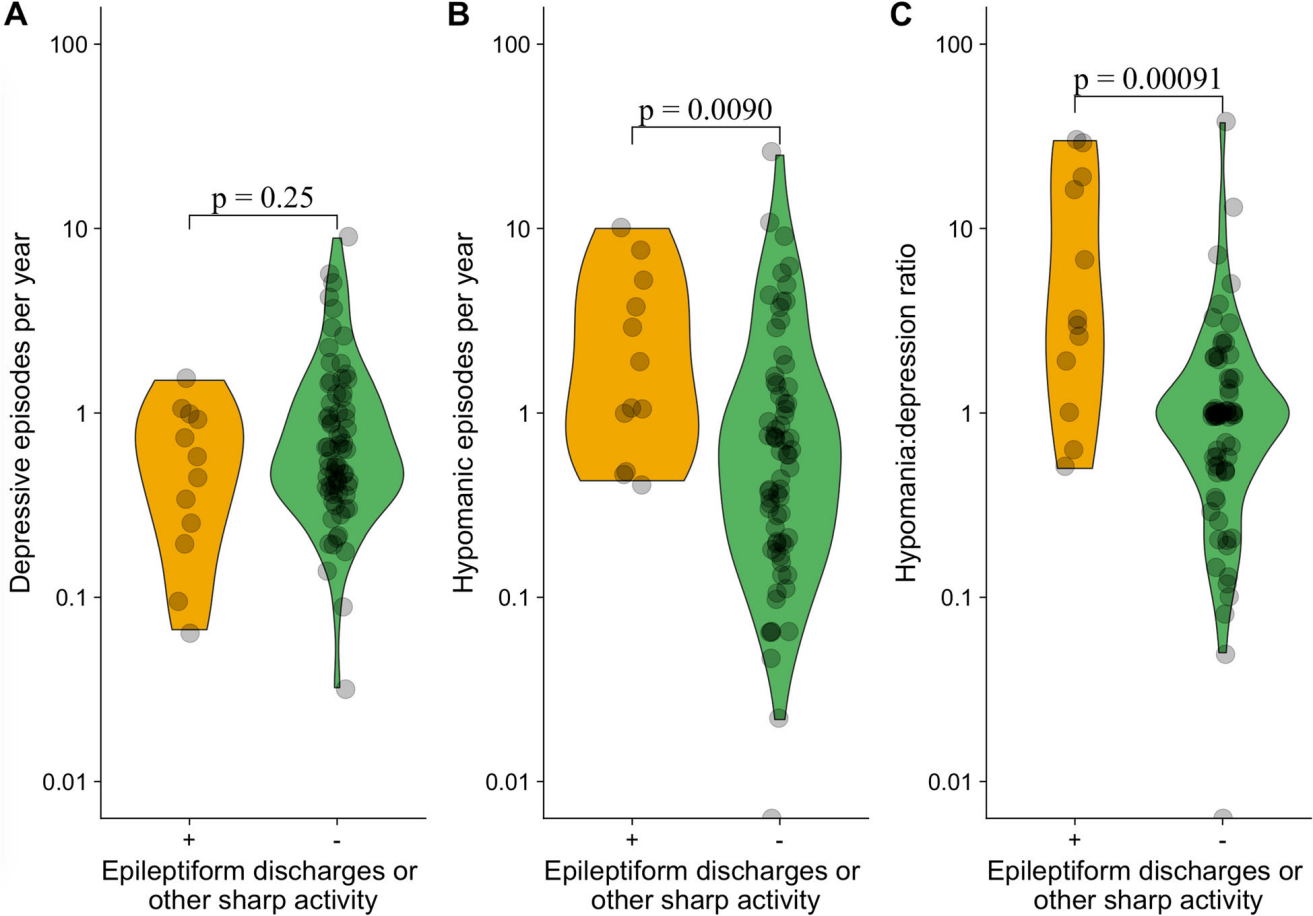
Frequency and ratio of depressive and hypomanic episodes among patients with and without epileptiform discharges or other sharp activity. Data are presented as violin plots with individual level data points at logarithmic y-axes. A small horizontal and vertical jitter is added to better discriminate overlying data points

Participants with epileptiform discharges or other sharp activity had significantly more hypomanic episodes per year (median (interquartile range (IQR)) 1.5 (3.2) vs. 0.61 (1.1), *p* = 0.0090) and a higher hypomania:depression ratio (median (IQR) 3.2 (16) vs. 1.0 (1.0), *p* = 0.00091) as compared to participants without (Table 2, Fig. 1). There were no significant differences between the groups on the other variables (Table 2).

**Table 2:** Course of illness among participants with and without epileptiform discharges or other sharp activity

	ED/SA+ (***n*** = 12)	ED/SA- (***n*** = 74)	***p***-value
Age at onset			0.95^†^
mean ± SD	19 ± 13	18 ± 11	
median (IQR)	15 (3.5)	15 (7.8)	
Depressive episodes per year			0.25^†^
mean ± SD	0.60 ± 0.45	1.1 ± 1.4	
median (IQR)	0.51 (0.72)	0.63 (0.78)	
Hypomanic episodes per year			**0.0090**^†^
mean ± SD	3.0 ± 3.2	1.6 ± 3.5	
median (IQR)	1.5 (3.2)	0.61 (1.1)	
Hypomania:depression ratio			**0.00091**^†^
mean ± SD	9.7 ± 11	1.9 ± 4.7	
median (IQR)	3.2 (16)	1.0 (1.0)	
Admissions per decade^a^			0.31^†^
mean (SD)	0.81 (1.5)	1.2 (1.9)	
median (IQR)	0.44 (0.76)	0.63 (1.4)	
Rapid cycling, n (%)^b^	6 (60)	27 (36)	0.18^‡^

Abbreviations: *ED* epileptiform discharges, *SA* other sharp activity, *IQR* interquartile range, *SD* standard deviation. Missing data: ^a^ 1 in ED/SA+ group, ^b^ 2 in ED/SA+ group. Tests: † Wilcoxon rank sum test, ‡ Fisher’s exact test

The finding of an increased hypomania:depression ratio remained significant in separate analyses in Sample 1 (median (IQR) 2.0 (11) vs. 1.0 (0.67), *p* = 0.031) and Sample 2 (median (IQR) 3.3 (17) vs. 1.3 (1.5), *p* = 0.027) (Supplementary Tables 2a and 2b, Supplementary figures 1a and 1b).

### Specification of epileptiform discharges or other sharp activity

Two participants (2.3%) had epileptic discharges (spikes and sharp-slow wave) and ten participants (12%) had other sharp activity. The findings were located at the right frontal region (F4) (*n* = 1), left and right frontal region (*n* = 1), left fronto-temporal region (*n* = 2), right temporal region (*n* = 1), left and right parietal region (P3, P4) (*n* = 1), left parietal region (*n* = 1), right central region (C4) (*n* = 1), Cz (*n* = 1), right occipital region (*n* = 1), generally distributed (*n* = 1), and without specification (*n* = 1).

### Risk of bias in self-reported data

Clinicians regarded the participants self-reported answers ‘very reliable’ in both groups (median (IQR) 1 (0) vs. 1 (0), *p* = 0.98).

## Discussion

We found that epileptiform discharges or other sharp activity were associated with more hypomanic episodes per year and an increased hypomania:depression ratio among patients diagnosed with bipolar II disorder in psychiatric care. The hypomania:depression ratio had a median value of 3.2:1 in the case group compared to 1:1 in the control group.

An increased hypomania/mania:depression ratio is previously reported in a sample of 20 patients with bipolar disorder due to traumatic brain injury where half of the patients had posttraumatic epilepsy [26]. In patients with bipolar II disorder in psychiatric care, however, Tondo et al. found a group level ratio of 1:1.9 [27], which approximates the median ratio of our control group. We have previously reported that the hypomania/mania:depression ratio was not higher among bipolar disorder patients with premorbid traumatic brain injury as compared to patients without [28]. Taken together, our and previous findings suggest that epileptiform discharges or other sharp activity per se, i.e. independently of a history of epileptic seizures and traumatic brain injury, could be associated with an increased hypomania:depression ratio among patients with bipolar II disorder.

We cannot use data from a small cross-sectional study to conclude on the causes of our findings. Still, one possible explanation is that the etiology of bipolar II disorder could differ between patients with and without epileptiform discharges or other sharp activity. Such differences could be related to genetic factors, as both bipolar disorder and epilepsy have high estimates of heritability [29, 30]. Results from a nation-wide registry-based study [31] and genome-wide association studies [32] suggest that there is no genetic correlation between bipolar disorder and epilepsy. However, traits without a genetic correlation can still be influenced by overlapping genetic variants due to a mixture of discordant and concordant effects on the traits of interest [33]. For example, a common genetic variant implicating the *ANK3* gene has consistently been associated with risk for bipolar disorder [34], and transgenic mice deficient for the related ANK3 exon 1b have a gene dosage-dependent phenotype with behavioral changes and epilepsy [35]. Also, a de novo missense mutation within *ANK3* is reported in a child with Lennox-Gastaut syndrome [36].

The prevalence of epileptiform discharges or other sharp activity in a sample of patients with bipolar II disorder in psychiatric care is to our knowledge not previously reported. Epileptiform discharges among 2.3% is higher than the 0.5% prevalence reported among healthy individuals [13], while the 12% prevalence of other sharp activity is in line with a previous study of patients hospitalized due to manic episodes [15].

Our study has limitations. Self-reported data are prone to recall bias, which, however, is unlikely to be related to findings among the participants referred to EEGs after clinical assessments. Lack of data on inter-rater reliability of diagnostic evaluations and assessment of EEGs could have introduced noise in our results. A standardized procedure for EEG recording and assessment (e.g. regarding duration of recordings, filtering of data, removal of artifacts, extraction of sub-bands, and classification of epileptiform discharges) could have increased the generalizability of our results. A single scalp EEG has a sensitivity for epileptiform discharges of about 50% in patients with epilepsy [37] and could be lower in our sample with high frequencies of treatment with anticonvulsants. The interplay between epileptiform discharges or other sharp activity, course of illness, and pharmacological treatment is difficult to untangle in a cross-sectional study. More valid data could possibly be obtained if the sample was restricted to medication-naïve participants included at their first clinical presentation. The small sample size increases the risk of type II statistical errors. The small sample size and lack of control for multiple comparisons also increase the risk of false positive findings, however; the main finding was significant in two independent samples and its *p*-value in the pooled sample would have survived Bonferroni correction for multiple comparisons.

## Conclusions

Among patients diagnosed with bipolar II disorder in psychiatric care, encephalographic epileptiform discharges or other sharp activity are associated with more hypomanic episodes per year and an increased hypomania:depression ratio independently of a history of epileptic seizures. If our results are replicated in a larger prospective study with comprehensive control of confounders, EEG findings could be of prognostic importance for patients with bipolar II disorder in psychiatric care.

## Supplementary Information

**Additional file 1: Supplementary note 1a.** Encephalographic recordings in Sample 1. **Supplementary note 1b.** Encephalographic recordings in Sample 2. **Supplementary Table 1a.** Characteristics of participants included in Sample 1. **Supplementary Table 1b.** Characteristics of participants included in Sample 2. **Supplementary Table 2a.** Course of illness among participants included in Sample 1. **Supplementary Table 2b.** Course of illness among participants included in Sample 2. **Supplementary figure 1a.** Frequency and ratio of depressive and hypomanic episodes among participants included in Sample 1. **Supplementary figure 1b.** Frequency and ratio of depressive and hypomanic episodes among participants included in Sample 2.

**Additional file 2.** STROBE Statement—checklist of items that should be included in reports of observational studies.

## Data Availability

The data that support the findings of this study are not publicly available due to the sensitive nature of the information. Anonymous summary data can be made available from corresponding author upon request. Restrictions apply to sharing the sensitive individual-level human data which are regulated by the approvals from Ethics and Data protection agency limiting the use of the data. Individual-level data can be shared if the necessary Ethics and Data protection approvals are obtained.

## Abbreviations

BRAIN: Bipolar Research and Innovation Network Norway
EEG: electroencephalogram
HCL-32: Hypomania Checklist
IDS-C30: Inventory of Depressive Symptoms Clinician Rated 30
M.I.N.I.: Mini-International Neuropsychiatric Interview
NEQ: Network Entry Questionnaire
SCID 1: Structured Clinical Interview for DSM IV Axis I Disorders
YMRS: Young Mania Rating Scale
