# Supplementary material for: Differences in course of illness between patients with bipolar II disorder with and without epileptiform discharges or other sharp activity on electroencephalograms: a cross-sectional study
Source: BMC Psychiatry. 2020 Dec 7;20:582. doi: 10.1186/s12888-020-02968-4 (PMC7720555; doi:10.1186/s12888-020-02968-4)
Supplement: Supplementary file 1 — Additional file 1: Supplementary note 1a. Encephalographic recordings in Sample 1. Supplementary note 1b. Encephalographic recordings in Sample 2. Supplementary Table 1a. Characteristics of participants included in Sample 1. Supplementary Table 1b. Characteristics of participants included in Sample 2. Supplementary Table 2a. Course of illness among participants included in Sample 1. Supplementary Table 2b. Course of illness among participants included in Sample 2. Supplementary figure 1a. Frequency and ratio of depressive and hypomanic episodes among participants included in Sample 1. Supplementary figure 1b. Frequency and ratio of depressive and hypomanic episodes among participants included in Sample 2. [file 12888_2020_2968_MOESM1_ESM.zip › 20201115_bd2_eeg_rev_supplementary_materialsR2.docx]

**Supplementary materials**

Authors:

O.K. Drange^1,2^, S.G. Sæther^2^, P.I. Finseth^1,2^, G. Morken^1,2^, A.E. Vaaler^1,2^, V. Arntsen^3^, O. Henning^4^, O.A. Andreassen^5,6^, T. Elvsåshagen^5,6,7^, U.F. Malt^5,8^, E. Bøen^9^

^1^ Department of Mental Health, Norwegian University of Science and Technology, Norway

^2^ Division of Mental Health, St. Olavs Hospital, Trondheim University Hospital, Norway

^3^ Department of Neurology and Clinical Neurophysiology, St. Olavs Hospital, Trondheim University Hospital, Norway

^4^ National Center for Epilepsy, Oslo University Hospital, Norway

^5^ Institute of Clinical Medicine, University of Oslo, Norway

^6^ NORMENT, Division of Mental Health and Addiction, Oslo University Hospital, Norway

^7^ Department of Neurology, Oslo University Hospital, Norway

^8^ Department of Research and Education, Division of Clinical Neuroscience, Oslo University Hospital, Norway

^9^ Psychosomatic and CL Psychiatry, Division of Mental Health and Addiction, Oslo University Hospital, Oslo, Norway

Corresponding author:

Ole Kristian Drange

Address St Olavs Hospital HF avd Østmarka, Postboks 3250 Torgarden, 7006 Trondheim, Norway

Phone +47 47623258

E-mail [ole.kristian.drange@gmail.com](mailto:ole.kristian.drange@gmail.com)

Table of content:

[Supplementary note 1a: Encephalographic recordings in Sample 1. 3](#_Toc53174855)

[Supplementary note 1b: Encephalographic recordings in Sample 2. 4](#_Toc53174856)

[Supplementary table 1a: Characteristics of participants included in Sample 1. 5](#_Toc53174857)

[Supplementary table 1b: Characteristics of participants included in Sample 2. 7](#_Toc53174858)

[Supplementary table 2a: Course of illness among participants included in Sample 1. 9](#_Toc53174859)

[Supplementary table 2b: Course of illness among participants included in Sample 2. 10](#_Toc53174860)

[Supplementary figure 1a: Frequency and ratio of depressive and hypomanic episodes among participants included in Sample 1. 11](#_Toc53174861)

[Supplementary figure 1b: Frequency and ratio of depressive and hypomanic episodes among participants included in Sample 2. 12](#_Toc53174862)

# Supplementary note 1a: Encephalographic recordings in Sample 1.

Routine electroencephalography with time-locked video were recorded with NicoletOne EEG system (Natus Medical Inc., CA, USA). Sampling rate was 256 Hz. The settings for the high-pass filter and low-pass filter were 0.5 Hz and 70 Hz respectively, 50 Hz notch filter was available. Silver chloride disc electrodes were attached on the scalp with a conductive paste. The skin was prepared with light abrasion to reduce impedance (contact resistance), and was kept less than 5kΩ to reduce the noise artefacts and other interference. Prerecording procedure with interelectrode impedance check was performed (1). Electrode placement is according to the international 10-20 system (2). Twenty-three scalp electrodes (including T1, T2 and Pg2), plus reference (placed between Fz and Cz), ground and two-channel electrocardiogram was used. Montages include longitudinal bipolar, transversal bipolar, laplacian and average referencial.

Routine EEG recording time was 20 minutes in resting condition with closed eyes. Activation procedures performed include hyperventilation, photic stimulation, eye opening, and eye closure, as well as eye movements. The duration of hyperventilation was 3 minutes, with a continued recording for 2-3 minutes after cessation of hyperventilation. Photic stimulation was performed with separate trains of photo flashes of 10 seconds duration for each frequency and with intervals of 7 second trains with the following frequencies: 2, 4, 6, 8, 10, 12, 14, 16, 18, 20, 60, 50, 40, 30 and 25 Hz. The total duration of photic activation time was 4 minutes.

# Supplementary note 1b: Encephalographic recordings in Sample 2.

EEGs were recorded with a NicoletOne system (Natus Medical Inc., CA, USA) using the standardized 25 electrode array of the IFCN (3) including the inferior temporal chain and a sampling frequency of 256 Hz. The nomenclature in the basic array is consistent with the 10-10-system. The recordings lasted 90 minutes. Approximately 5 minutes after the recording was started participants hyperventilated for 3 minutes, followed by a rest period of 5 minutes. Then intermittent photic stimulation was performed with a protocol of 10 seconds of stimulation with increasing frequencies (6Hz, 8Hz, 10Hz, 12Hz, 14Hz, 16Hz, 18Hz, 20Hz, 22Hz, 24Hz) where participants for a few seconds opened their eyes during stimulation followed by stimulation with decreasing frequencies (24Hz, 22Hz, 20Hz, 18Hz, 16Hz, 14Hz, 12Hz, 10Hz, 8Hz, 6Hz, 4Hz, 2Hz) while participants kept their eyes closed. During the rest of the EEG registration the participants were asked to lie relaxed with closed eyes.

# Supplementary table 1a: Characteristics of participants included in Sample 1.

|  | ED/SA+  (n=7) | ED/SA-  (n=57) | p-value |
| --- | --- | --- | --- |
|  |  |  |  |
| Age  mean ± SD  median (IQR) | 35 ± 19  28 (24) | 39 ± 12  40 (22) | 0.37^†^ |
| Women, n (%) | 3 (43) | 28 (49) | 1.0^‡^ |
| Years of education  mean ± SD  median (IQR) | 5.3 ± 3.1  5.0 (3.5) | 4.3 ± 3.2  4.0 (4.0) | 0.36^†^ |
| Family history, n (%)  Schizophrenia^a^  Bipolar disorder^b^  Depression^c^ | 0 (0)  2 (29)  2 (29) | 4 (7.5)  11 (20)  22 (39) | 1.0^‡^  0.63^‡^  0.70^‡^ |
| Medical history, n (%) |  |  |  |
| Migraine^d^ | 1 (14) | 7 (13) | 1.0^‡^ |
| Traumatic brain injury^d^ | 0 (0) | 7 (13) | 1.0^‡^ |
| Epileptic seizures | 0 (0) | 2 (3.5) | 1.0^‡^ |
| Pharmacological treatm., n (%) |  |  |  |
| Lithium | 0 (0) | 2 (3.5) | 1.0^‡^ |
| Anticonvulsants | 6 (86) | 48 (84) | 1.0^‡^ |
| Antipsychotics | 4 (57) | 27 (47) | 0.70^‡^ |
| Antidepressants | 4 (57) | 26 (46) | 0.70^‡^ |
| Anxiolytics/hypnotics | 3 (43) | 20 (35) | 0.69^‡^ |
| None | 0 (0) | 1 (1.8) | 1.0^‡^ |
| Psychiatric comorbidity, n (%) |  |  |  |
| Alcohol harmful use or dependence | 0 (0) | 7 (12) | 1.0^‡^ |
| Drugs harmful use or dependence | 0 (0) | 4 (7.0) | 1.0^‡^ |
| Agoraphobia | 0 (0) | 1 (1.8) | 1.0^‡^ |
| Social phobia | 0 (0) | 3 (5.3) | 1.0^‡^ |
| Specific phobia | 0 (0) | 0 (0) | 1.0^‡^ |
| Panic disorder | 0 (0) | 1 (1.8) | 1.0^‡^ |
| Obsessive-compulsive disorder | 0 (0) | 0 (0) | 1.0^‡^ |
| Symptom burden |  |  |  |
| IDS-C_30_ score |  |  |  |
| mean ± SD | 24 ± 16 | 27 ± 10 | 0.34^†^ |
| median (IQR) | 25 (19) | 28 (14) |  |
| YMRS score |  |  | 0.55^†^ |
| mean ± SD | 0.86 (1.6) | 1.7 (2.9) |  |
| median (IQR) | 0.0 (1.0) | 0.0 (3.0) |  |

Abbreviations: ED epileptiform discharges, SA other sharp activity, IDS-C30 Inventory of Depressive Symptoms Clinician Rated, IQR interquartile range, SD standard deviation, YMRS Young Mania Rating Scale. Missing data: ^a^ 4 in ED/SA- group, ^b^ 2 in ED/SA- group, ^c^ 1 in ED/SA- group, ^d^ 1 in ED/SA- group. Tests: † Wilcoxon rank sum test, ‡ Fisher’s exact test.

# Supplementary table 1b: Characteristics of participants included in Sample 2.

|  | ED/SA+  (n=5) | ED/SA-  (n=17) | p-value |
| --- | --- | --- | --- |
|  |  |  |  |
| Age  mean ± SD  median (IQR) | 34 ± 4.5  34 (4.0) | 32 ± 7.1  32 (10) | 0.84^†^ |
| Women, n (%) | 5 (100) | 12 (71) | 0.29^‡^ |
| Years of education  mean ± SD  median (IQR) | 6.6 ± 3.4  7.0 (3.0) | 6.2 ± 3.4  6.0 (6.0) | 0.87^†^ |
| Family history, n (%)  Schizophrenia  Bipolar disorder  Depression | 0 (0)  1 (20)  3 (60) | 1 (5.9)  2 (12)  17 (100) | 1.0^‡^  1.0^‡^  0.043^‡^ |
| Medical history, n (%) |  |  |  |
| Migraine | 2 (40) | 4 (24) | 0.59^‡^ |
| Traumatic brain injury | 1 (20) | 4 (24) | 1.0^‡^ |
| Epileptic seizures | 0 (0) | 1 (5.9) | 1.0^‡^ |
| Pharmacological treatm., n (%)^a^ |  |  |  |
| Lithium | 0 (0) | 0 (0) | 1.0^‡^ |
| Anticonvulsants | 2 (50) | 14 (82) | 0.23^‡^ |
| Antipsychotics | 0 (0) | 2 (12) | 1.0^‡^ |
| Antidepressants | 1 (25) | 8 (47) | 0.60^‡^ |
| Anxiolytics/hypnotics | 0 (0) | 4 (24) | 0.55^‡^ |
| None | 1 (25) | 1 (5.9) | 0.35^‡^ |
| Psychiatric comorbidity, n (%) |  |  |  |
| Alcohol harmful use or dependence | 0 (0) | 3 (18) | 1.0^‡^ |
| Drugs harmful use or dependence | 0 (0) | 0 (0) | 1.0^‡^ |
| Agoraphobia | 0 (0) | 2 (12) | 1.0^‡^ |
| Social phobia | 1 (20) | 5 (29) | 1.0^‡^ |
| Specific phobia | 1 (20) | 0 (0) | 0.23^‡^ |
| Panic disorder | 3 (60) | 11 (65) | 1.0^‡^ |
| Obsessive-compulsive disorder | 1 (20) | 1 (5.9) | 0.41^‡^ |
| Attention deficit hyperactivity disorder | 0 (0) | 1 (5.9) | 1.0^‡^ |
| Symptom burden |  |  |  |
| IDS-C_30_ score |  |  |  |
| mean ± SD | 23 ± 5.7 | 18 ± 9 | 0.19^†^ |
| median (IQR) | 24 (9.0) | 15 (15) |  |
| YMRS score |  |  | 0.41^†^ |
| mean ± SD | 3.0 (2.7) | 4.1 (2.8) |  |
| median (IQR) | 2.0 (2.0) | 3.0 (3.0) |  |

Abbreviations: ED epileptiform discharges, SA other sharp activity, IDS-C30 Inventory of Depressive Symptoms Clinician Rated, IQR interquartile range, SD standard deviation, YMRS Young Mania Rating Scale. Missing data: ^a^ 1 in ED/SA+ group. Tests: † Wilcoxon rank sum test, ‡ Fisher’s exact test.

# Supplementary table 2a: Course of illness among participants included in Sample 1.

|  | ED/SA+  (n=7) | ED/SA-  (n=57) | p-value |
| --- | --- | --- | --- |
| Age at onset  mean ± SD  median (IQR) | 22 ± 17  16 (5.5) | 19 ± 12  16 (5.5) | 0.80^†^ |
| Depressive episodes per year  mean ± SD  median (IQR) | 0.63 ± 0.41  0.71 (0.63) | 0.66 ± 0.53  0.48 (0.58) | 0.76^†^ |
| Hypomanic episodes per year  mean ± SD  median (IQR) | 2.5 ± 3.5  0.95 (2.0) | 0.59 ± 0.77  0.36 (0.55) | **0.012**^†^ |
| Hypomania:depression ratio  mean ± SD  median (IQR) | 8.3 ± 11  2.0 (11) | 1.0 ± 1.0  1.0 (0.67) | **0.031**^†^ |
| Admissions per decade  mean (SD)  median (IQR) | 1.4 (1.8)  0.74 (0.87) | 1.5 (2.0)  0.82 (1.4) | 0.75^†^ |
| Rapid cycling, n (%)^a^ | 1 (20) | 11 (19) | 1.0^‡^ |

Abbreviations: ED epileptiform discharges, SA other sharp activity, IQR interquartile range, SD standard deviation. Missing data: ^a^ 2 in ED/SA+ group. Tests: † Wilcoxon rank sum test, ‡ Fisher’s exact test.

# Supplementary table 2b: Course of illness among participants included in Sample 2.

|  | ED/SA+  (n=5) | ED/SA-  (n=17) | p-value |
| --- | --- | --- | --- |
| Age at onset^a^  mean ± SD  median (IQR) | 14 ± 1.9  14 (2.0) | 14 ± 4.1  14 (4.0) | 0.91^†^ |
| Depressive episodes per year  mean ± SD  median (IQR) | 0.55 ± 0.54  0.36 (0.16) | 2.5 ± 2.3  1.5 (2.9) | **0.011**^†^ |
| Hypomanic episodes per year  mean ± SD  median (IQR) | 3.7 ± 2.9  3.7 (3.9) | 4.9 ± 5.9  3.3 (4.0) | 0.97^†^ |
| Hypomania:depression ratio  mean ± SD  median (IQR) | 12 ± 13  3.3 (17) | 4.5 ± 9.0  1.3 (1.5) | **0.027**^†^ |
| Admissions per decade  mean (SD)  median (IQR) | 0.11 (0.24)  0 (0) | 0.074 (0.30)  0 (0) | 0.43^†^ |
| Rapid cycling, n (%) | 5 (100) | 16 (94) | 1.0^‡^ |

Abbreviations: ED epileptiform discharges, SA other sharp activity, IQR interquartile range, SD standard deviation. Tests: † Wilcoxon rank sum test, ‡ Fisher’s exact test.

# Supplementary figure 1a: Frequency and ratio of depressive and hypomanic episodes among participants included in Sample 1.


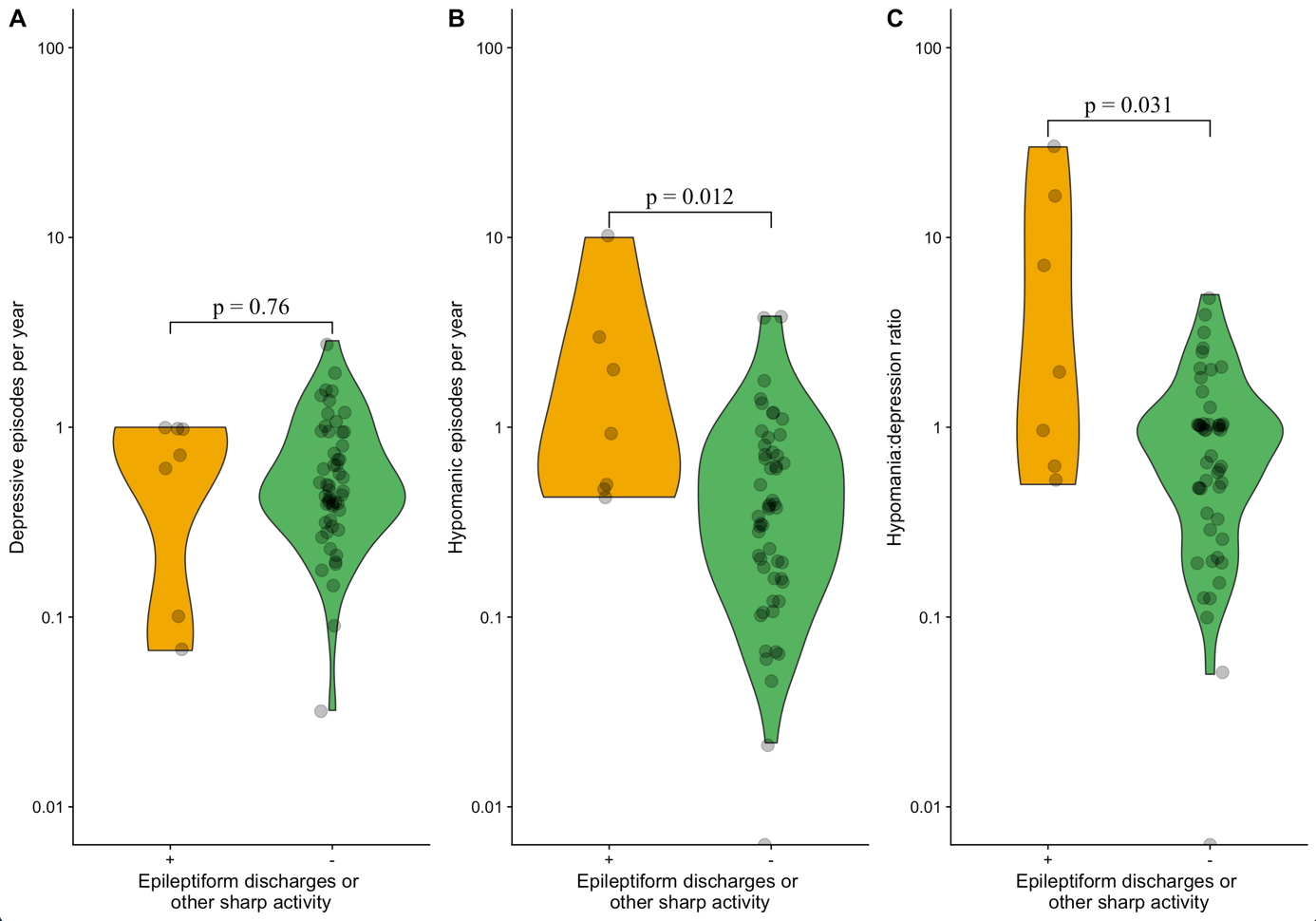
Data are presented as violin plots with individual level data points at logarithmic y-axes. A small horizontal and vertical jitter is added to better discriminate overlying data points.

# Supplementary figure 1b: Frequency and ratio of depressive and hypomanic episodes among participants included in Sample 2.


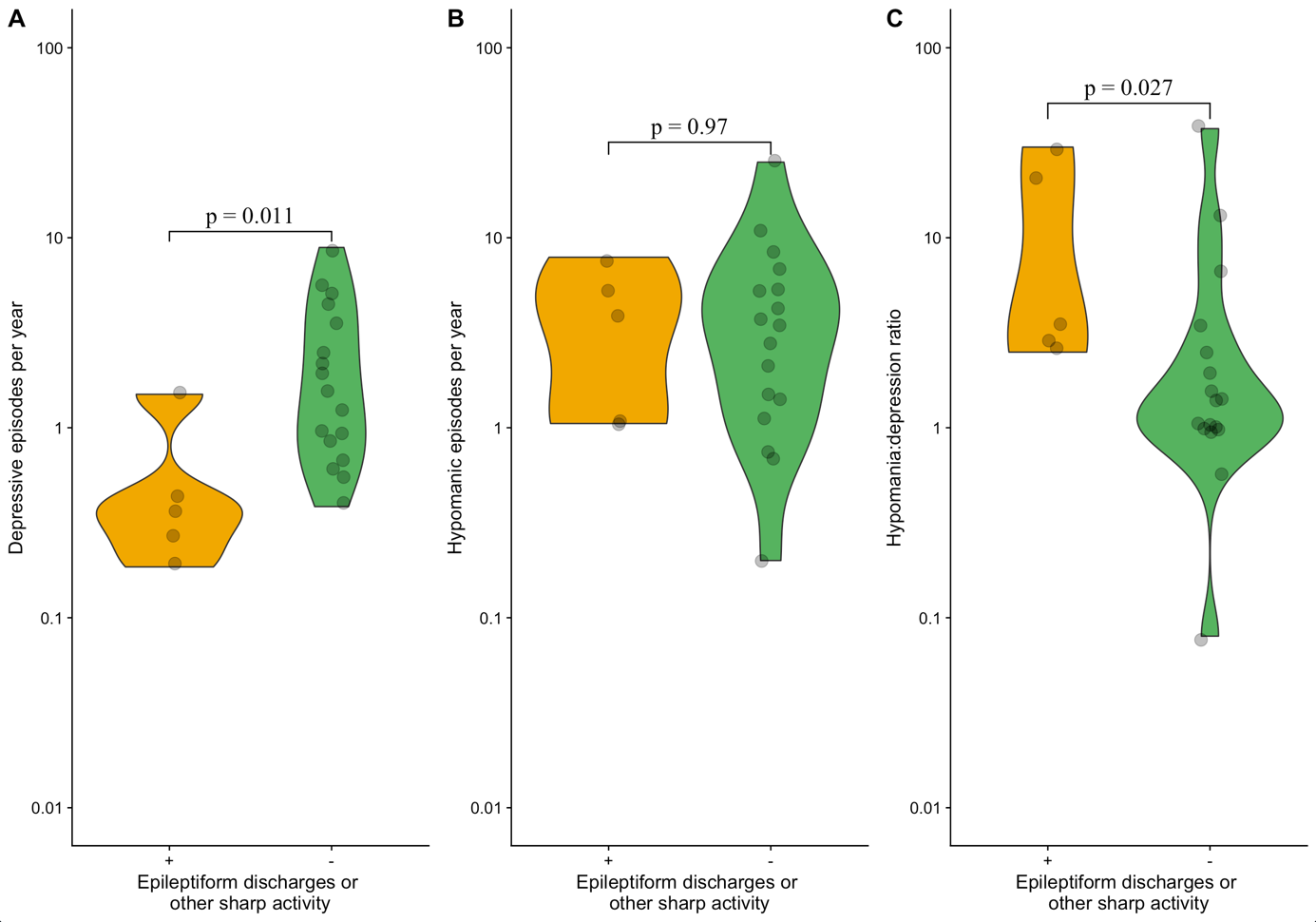


Data are presented as violin plots with individual level data points at logarithmic y-axes. A small horizontal and vertical jitter is added to better discriminate overlying data points.

Supplementary references:

1. Acharya JN, Hani A, Cheek J, Thirumala P, Tsuchida TN. American Clinical Neurophysiology Society Guideline 2: Guidelines for Standard Electrode Position Nomenclature. J Clin Neurophysiol. 2016;33:308–11.

2. Sinha SR, Sullivan L, Sabau D, San-Juan D, Dombrowski KE, Halford JJ, et al. American Clinical Neurophysiology Society Guideline 1: Minimum Technical Requirements for Performing Clinical Electroencephalography. J Clin Neurophysiol. 2016;33:303–7.

3. Seeck M, Koessler L, Bast T, Leijten F, Michel C, Baumgartner C, et al. The standardized EEG electrode array of the IFCN. Clin Neurophysiol. 2017;128:2070–7.
