# Supplementary figures and images for: Differences in course of illness between patients with bipolar II disorder with and without epileptiform discharges or other sharp activity on electroencephalograms: a cross-sectional study
Source: BMC Psychiatry. 2020 Dec 7;20:582. doi: 10.1186/s12888-020-02968-4 (PMC7720555; doi:10.1186/s12888-020-02968-4)

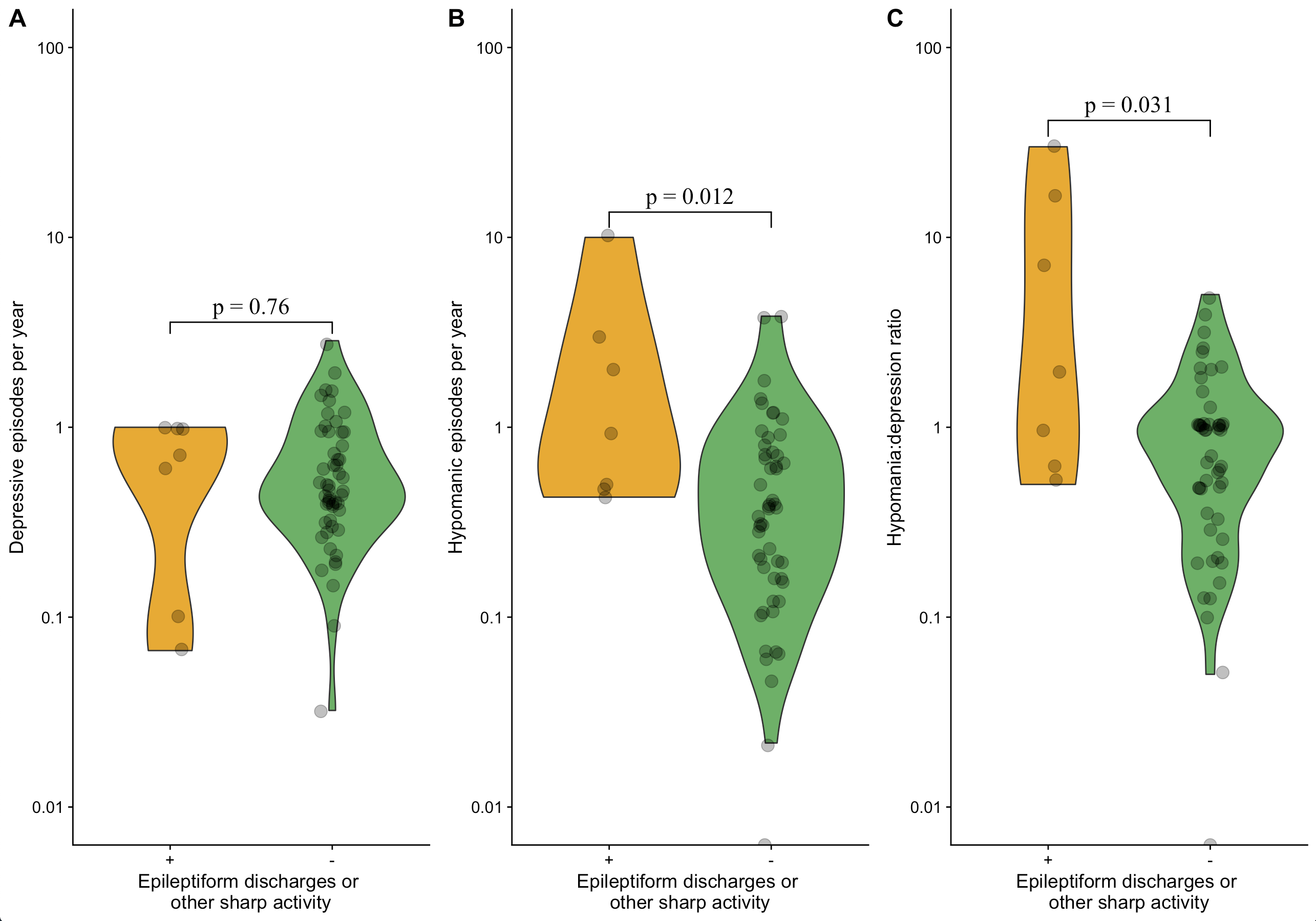

Supplement: Supplementary file 1 — Additional file 1: Supplementary note 1a. Encephalographic recordings in Sample 1. Supplementary note 1b. Encephalographic recordings in Sample 2. Supplementary Table 1a. Characteristics of participants included in Sample 1. Supplementary Table 1b. Characteristics of participants included in Sample 2. Supplementary Table 2a. Course of illness among participants included in Sample 1. Supplementary Table 2b. Course of illness among participants included in Sample 2. Supplementary figure 1a. Frequency and ratio of depressive and hypomanic episodes among participants included in Sample 1. Supplementary figure 1b. Frequency and ratio of depressive and hypomanic episodes among participants included in Sample 2. [file 12888_2020_2968_MOESM1_ESM.zip › suppl_figure_1a.png]

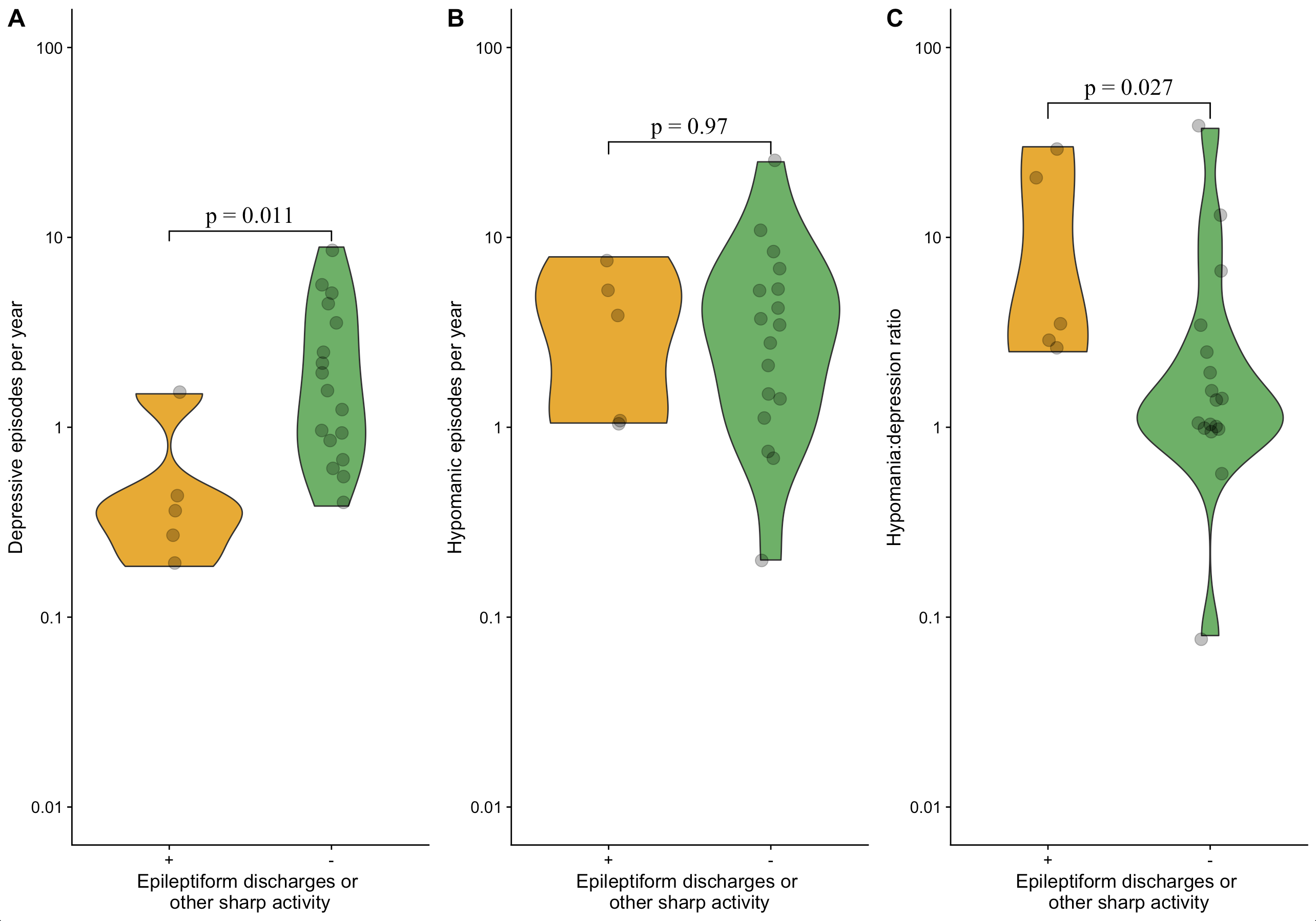

Supplement: Supplementary file 1 — Additional file 1: Supplementary note 1a. Encephalographic recordings in Sample 1. Supplementary note 1b. Encephalographic recordings in Sample 2. Supplementary Table 1a. Characteristics of participants included in Sample 1. Supplementary Table 1b. Characteristics of participants included in Sample 2. Supplementary Table 2a. Course of illness among participants included in Sample 1. Supplementary Table 2b. Course of illness among participants included in Sample 2. Supplementary figure 1a. Frequency and ratio of depressive and hypomanic episodes among participants included in Sample 1. Supplementary figure 1b. Frequency and ratio of depressive and hypomanic episodes among participants included in Sample 2. [file 12888_2020_2968_MOESM1_ESM.zip › suppl_figure_1b.png]
